# Effects of competitive sports on temporomandibular dysfunction: a literature review

**DOI:** 10.1007/s00784-020-03742-2

**Published:** 2020-12-26

**Authors:** Hannah Charlotte Freiwald, Nico Peter Schwarzbach, Anne Wolowski

**Affiliations:** Poliklinik für Prothetische Zahnmedizin und Biomaterialien, Albert-Schweitzer-Campus 1/W30, Westfälische Wilhelms-Universität Münster, 48149 Munster, Germany

**Keywords:** Temporomandibular dysfunctions, TMD-prevalence, Competitive sports, Sports

## Abstract

**Objectives:**

The German Society of Craniomandibular Function and Disorders recommends that patients suffering from temporomandibular dysfunctions should practice sports in order to compensate for everyday stress. This raises the question as to what extent competitive athletes develop temporomandibular dysfunctions or whether their athletic activities protect them. With the present literature review, the authors intend to give an overview of the currently available publications on this topic.

**Materials and methods:**

A literature research in the PubMed and Google Scholar databases was performed to filter out the currently available publications on the topic ‚sports, and temporomandibular dysfunction.

**Results:**

Out of 114 available articles, seven met the inclusion criteria. Two other relevant articles were found in the list of references, so that in total, nine publications were picked for the review. In case numbers ranging from eight to 347 subjects, a temporomandibular dysfunction was detected with a prevalence between 11.7% and 100% for athletes and between 11.11% and 14.3% for non-athletes. Different kinds of sports were evaluated, all of them contact sports: basketball, handball, wrestling, boxing, karate, mixed martial arts, field hockey, water polo, and soccer. One study compared athletes with and without consumption of anabolic steroids, regardless of the type of sport. The level of athletic performance varied across the different studies.

**Conclusions:**

Currently, studies dealing with the effect of competitive sports on temporomandibular dysfunction are scarce. Inconsistent methodological procedures permit only limited comparability.

**Clinical relevance:**

A general trend, however, can already be discerned: professional athletes suffer from temporomandibular dysfunctions more frequently than non-athletes.

## Objectives

Temporomandibular dysfunctions (TMD) include specific functional problems that can affect the masticatory muscles, the temporomandibular joints, and related structures [1]. Among the varied range of symptoms are pain in the area of the masticatory muscles or temporomandibular joints, crepitations, disk displacements, and restrictions or asymmetries in the movements of the lower jaw [1, 2].

Information on the prevalence in the general population varies depending on the study and the method of investigation. Barbosa et al. [3] found a TMD with a frequency of 39.3%, while Heß [4] reported a prevalence of 17.1%. In the third German oral health study [5], 21.3% of the adults were diagnosed with TMD based on their medical history and 51.1% were diagnosed based on clinical examination.

The German Society of Craniomandibular Function and Disorders recommends that patients suffering from TMD should engage in physical activity to help compensate for everyday stress [6]. Especially endurance sport is referred to in this context [6]. This raises the question as to what extent people whose lives are focused on (competitive) sports have TMD or are protected from it by their athletic activity. Competitive athletes are forced to organize their social life, education, or job as efficiently as possible to fit with their daily training workload. Early on, young athletes learn how to work in a disciplined and structured way in order to be successful in both sports and school. Does this double burden entail a higher risk of TMD or do competitive athletes create the necessary compensation for everyday life through their training?

The present literature review aims to address these matters from a scientific perspective.

## Materials and methods

### Inclusion criteria

The studies included in this review investigated TMD or symptoms of TMD in competitive athletes. Due to the small number of publications on this topic, studies in which information on the athletic performance level is missing were included as well.

Studies featuring a control group consisting of non-athletes were preferred. However, due to the limited number of publications, studies in which the control group consisted of athletes of a different kind of sport and studies with no control group at all were also included in this review.

All prospective or retrospective studies, cohort studies, case control studies, and cross-sectional studies were accepted.

### Search strategy

The databases of PubMed and Google Scholar were searched using the following query: “((serious sport) OR (high performance sport) OR (competitive sport)) AND ((temporomandibular disorder) OR (TMD)) and (sport) AND temporomandibular disorder.” All publications until April 2020 were considered.

First, the titles of all search results were scanned by one author and publications unavailable in English or German, as well as those with irrelevant topics, were excluded. Two authors then read the abstracts of the remaining studies independently. Based on these abstracts, the potentially relevant articles were selected and the full texts were read. If these articles contained references to other possibly relevant literature, abstracts and, if necessary, full texts were read as well. In the end, both authors compared their list of relevant articles. In cases of disagreement regarding the relevance of an article, in- or exclusion was decided after a discussion of the full text.

## Results

**Fig. 1.**
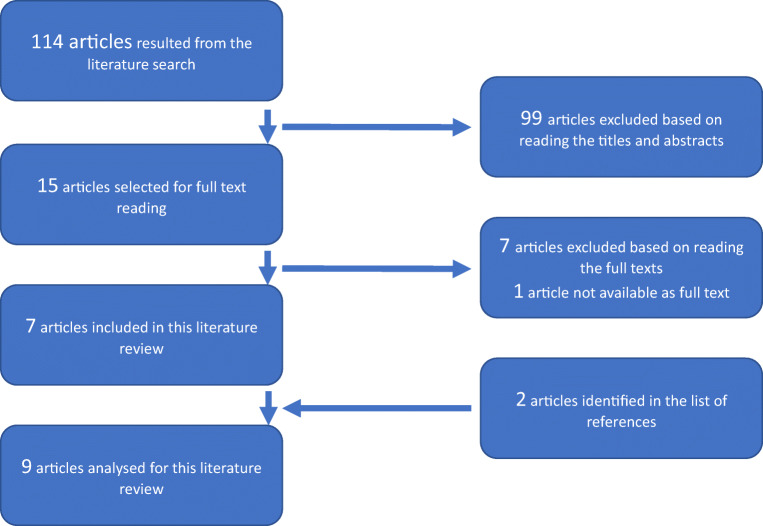
Procedure of the literature search

Based on the search terms, a total amount of 114 available articles resulted, 99 of which were excluded after reading the titles and abstracts. The full texts of 14 studies were obtained; for one article, the full text was not retrievable. Since seven of the full-text articles did not meet the inclusion criteria, only data from the remaining seven studies was included in the evaluation. While reading the full texts, two other relevant publications were identified in the list of references. In total, 9 studies were analyzed for the present review. Figure 1 shows the procedure of the literature search.

The articles included are six cohort studies [7–12] and three cross-sectional studies [13–15].

Different kinds of sport were taken into account, all of them contact sports: basketball [8, 9], handball [9, 11], wrestling [10], boxing [11], karate [12], mixed martial arts [12], field hockey [13], water polo [14], and soccer [15]. Only one study provided no information on the exercised sport but made a subdivision based on the consumption of anabolic steroids [7]. Table 1 shows the training workload and the athletic level of the respective groups.

**Table 1.** Overview of the general training data

First author [reference]	Type of sport	Age (in years)	Athletic level	Workload
Barros [7]	N/A	23 - 25	N/A	N/A
Weiler [8]	basketball	10 - 13	squad athletes	10 h/week
Weiler [9]	basketball/handball	10 - 18	squad athletes	10 h/week
Persson [10]	wrestling	16 - 34	N/A	N/A
Mendoza-Puente [11]	boxing/handball	18 - 35	competitive athletes	5 d/week
Bonotto [12]	karate	28.3	competitive athletes	11,8 h/week
	karate	24.7	recreational athletes	7,8 h/week
	MMA	24.1	competitive athletes	7,4 h/week
Zamora-Olave [13]	field hockey	14.1	N/A	N/A
Zamora-Olave [14]	water polo	15.0	amateurs to competitive athletes	N/A
Gay-Escoda [15]	soccer	21.0	competitive athletes	4 - 5 d/week 8 - 12 h/week

There was no consistent methodological approach in the included studies. While some examined the subjects and documented the results using the Helkimo index [7, 11] or the RDC/TMD [12], most of the studies used self-assessment questionnaires [8–10, 13–15], in some cases supplemented by a clinical examination [8–10, 15]. One study was based entirely on the results of clinical examination [11].

The case numbers ranged from eight to 347 subjects [7–15].

Three studies lacked control groups [13–15]. In three studies, the control group consisted of clearly defined non-athletes [8, 9, 12]. Three studies compared athletes from different sports [10–12]. Only one study compared athletes with and without the consumption of anabolic steroids regardless of the type of sport [7] (Table 2).

**Table 2.** Overview of the included studies

First author (year of publication) [reference]	Type of study	Level of evidence	Case number	Examined group incl. type of sport	Methodological approach	TMD-prevalence
Barros (2008) [7]	cohort study	III	n = 8	4 male athletes using anabolic steroids 4 male athletes not using anabolic steroids	anamnestic and clinical Helkimo Index	steroids group: 100% control group: 25%
Weiler (2010) [8]	cohort study	III	n = 87	46 male basketball players 41 male non-athletes	questionnaire; supplementary functional analysis if at least one finding was positive	basketball players: 26% non-athletes: 12%
Weiler (2013) [9]	cohort study	III	n = 161	49 female basketball players 40 female handball players 72 female non-athletes	questionnaire; supplementary functional analysis if at least one finding was positive	female athletes: 16.85% female non-athletes: 11.11%
Persson (1994) [10]	cohort study	III	n = 52	26 male wrestlers 26 male non-wrestlers	questionnaire; clinical examination of the stomatognathic system	no overall prevalence stated, prevalence of individual symptoms only
Mendoza-Puente (2014) [11]	cohort study	III	n = 38	18 male boxers 20 male handball players	clinical Helkimo Index	boxers: 77.77% at least moderate TMD handball players: 45.00% at least moderate TMD athletes: 60.53% at least moderate TMD
Bonotto (2016) [12]	cohort study	III	n = 82	24 competitive karatekas (7 f, 17 m) 17 amateur karatekas (4 f, 13 m) 13 competitive mixed martial arts athletes (3 f, 10 m) 28 non-athletes (8 f, 20 m)	portuguese version of RDC/TMD	competitive karatekas: 54.2% amateur karatekas: 17.6% competitive mixed martial arts athletes: 61.5% non-athletes: 14.3%
Zamora-Olave (2019) [13]	cross-sectional study	III	n = 325	325 field hockey players (91 f, 234 m)	questionnaire	field hockey players: 11.7%
Zamora-Olave (2018) [14]	cross-sectional study	III	n = 347	347 water polo players (123 f, 224 m)	questionnaire	water polo players: 20.2%
Gay-Escoda (2011) [15]	cross-sectional study	III	n = 30	30 male professional soccer players	anamnestic and clinical examination	no overall prevalence stated, prevalence of individual symptoms only
f: female; m: male						

The different studies reported TMD frequencies between 11.7% [13] and 100% [7] for athletes and 11.11% [9] and 14.3% [12] for non-athletes.

By means of anamnestic and clinical Helkimo index, Barros et al. [7] found that among athletes using anabolic steroids (four subjects), 25% showed mild signs of TMD (A_i_I and D_i_I) and 75% showed moderately severe symptoms (A_i_II and D_i_II). The control group of four undrugged athletes was 75% TMD-free (A_i_O and D_i_O) and only one athlete (25%) showed mild signs (A_i_I and D_i_I). The most common symptoms reported among the anabolic steroid users were trismus and pain in the masticatory muscles [7].

Weiler et al. [8] used a questionnaire to screen male basketball players and a control group of non-athletes for TMD symptoms (pain in the temporomandibular joint when chewing, headaches of unknown origin more than once a week, stiffness or fatigue in the temporomandibular joint, problems opening the mouth, bruxism and crepitations in the temporomandibular joint). If at least one of the findings was positive, an additional functional analysis (mobility of the jaw, crepitations of the temporomandibular joint (TMJ), pain during mandibular movements, tenderness on palpation of the TMJ, and the masticatory muscles) was carried out [8]. The authors [8] found temporomandibular dysfunctions in 26% (12/46 subjects) of the basketball players and 12% (5/41 subjects) of the non-athletes but reported no statistically significant difference between the groups in that regard. For both basketball players and non-athletes, the most common symptom was tenderness on palpation of the masticatory muscles (17.4% (8/46) and 7.3% (3/41)) [8].

Using the same study design but including female subjects (basketball and handball players as well as non-athletes), a subsequent study revealed a TMD frequency of 16.85% (15/89 subjects) for the female athletes group and 11.11% (8/72 subjects) for the non-athletes [9]. This difference was also not statistically significant [9]. According to Weiler et al. [9], tenderness on palpation of the masticatory muscles was the predominant symptom in both groups (5.62% (5/89) and 5.56% (4/72)).

Persson et al. [10] determined the prevalence of individual symptoms of temporomandibular dysfunction in wrestlers and non-wrestlers using a questionnaire and a clinical examination (palpation of the masseter and temporalis muscles, swelling of the temporomandibular joints, crepitations in the joints, pain during mandibular movements, mandibular deviation, maximum possible jaw opening, distance between retruded contact position, and maximal intercuspal position); an overall prevalence was not stated. The most frequent symptoms identified in the questionnaire were crepitations of the TMJ (15.38% or 4/26 subjects) in the wrestler group and headache as well as crepitations (3.85% or 1/26 subjects, respectively) in the control group [10]. Clinically, the most common symptom of TMD in both wrestlers and non-wrestlers was mandibular deviation (11.54% (3/26) and 19.23% (5/26)) [10].

Using the clinical Helkimo index, Mendoza-Puente et al. [11] detected at least moderate temporomandibular dysfunction (D_i_II) in 14 out of 18 boxers (77.77%) and nine out of 20 handball players (45.00%). In summary, these values indicated a TMD frequency in athletes (boxers and handball players) of 60.53% (23/38 subjects) [11]. Data on the frequency of individual symptoms was not provided in this study [11].

Using the RDC/TMD, Bonotto et al. [12] diagnosed Axis I temporomandibular dysfunctions in 54.2% (13/24 subjects) of competitive karatekas, 17.6% (3/17 subjects) of amateur karatekas, 61.5% (8/13 subjects) of competitive mixed martial arts athletes, and 14.3% (4/28 subjects) of non-athletes. The most common symptom in all four groups was dislocation of the disk (45.8% (11/24), 11.8% (2/17), 38.5% (5/13), and 7.1% (2/28)), while tenderness on palpation of the masticatory muscles was equally prevalent in the group of non-athletes (7.1% (2/28)) [12].

Zamora-Olave et al. [13] found a prevalence of temporomandibular dysfunctions of 11.7% (38/325 subjects) in field hockey players by means of a questionnaire (training workload, orofacial injuries including acute pain in the temporomandibular joint or masticatory muscles with aggravation during mandibular movements, use of a mouthguard). The authors did not provide additional information on the frequency of individual symptoms [13].

In a similarly designed study, Zamora-Olave et al. [14] used the same questionnaire to detect TMD among water polo players with a prevalence of 20.2% (70/347 subjects). Again, no information on the frequency of individual symptoms was included in this study [14].

Based on anamnestic information and clinical examination, Gay-Escoda et al. [15] showed that bruxism was the most common symptom (30% or 9/30 subjects) of temporomandibular dysfunction in professional soccer players. This study did not provide information on the overall TMD prevalence [15].

Tables 3 and 4 show the frequencies of individual symptoms reported in the respective studies.

**Table 3.** Frequency of symptoms among all subjects

	First author [reference]																
	Barros [7]		Weiler [8]		Weiler [9]		Persson [10]		Mendoza-Puente [11]		Bonotto [12]				Zamora- Olave [13]	Zamora- Olave [14]	Gay-Escoda [15]
	Anabolic steroids	No anabolic steroids	Basketball players	Non-athlete	Basketball/ Handball players	Non-athletes	Wrestlers	Nonwrestlers	Boxers	Handball players	Competitive karatekas	Amateur karatekas	Competitive MMA athletes	Non-athletes	Field hockey players	Water polo players	Pro soccer players
**TMD-symptoms (prevalence among all subjects in %):**																	
Tenderness on palpation of the masticatory muscles	N/A	N/A	17.4	7.3	5.62	5.56	N/A	N/A	N/A	N/A	12.5	5.9	30.8	7.1	N/A	N/A	N/A
Bruxism	N/A	N/A	13.0	0.0	4.49	1.39	N/A	N/A	N/A	N/A	N/A	N/A	N/A	N/A	N/A	N/A	30.0
Otalgia	N/A	N/A	6.5	2.4	3.37	1.39	N/A	N/A	N/A	N/A	N/A	N/A	N/A	N/A	N/A	N/A	N/A
Ear noise	N/A	N/A	6.5	2.4	2.25	1.39	N/A	N/A	N/A	N/A	N/A	N/A	N/A	N/A	N/A	N/A	N/A
Unilateral chewing	N/A	N/A	2.2	4.9	3.37	1.39	N/A	N/A	N/A	N/A	N/A	N/A	N/A	N/A	N/A	N/A	N/A
Mandibular deviation	N/A	N/A	6.5	2.4	1.12	2.78	11.54	19.23	N/A	N/A	N/A	N/A	N/A	N/A	N/A	N/A	6.7
headache	N/A	N/A	6.5	2.4	3.37	1.39	3.85	3.85	N/A	N/A	N/A	N/A	N/A	N/A	N/A	N/A	N/A
Crepitations of the TMJ	N/A	N/A	4.3	2.4	4.49	2.78	15.38 (a) 3.85 (c)	3.85 (a) 11.54 (c)	N/A	N/A	N/A	N/A	N/A	N/A	N/A	N/A	16.7
Disc displacement	N/A	N/A	N/A	N/A	N/A	N/A	N/A	N/A	N/A	N/A	45.8	11.8	38.5	7.1	N/A	N/A	N/A
Tenderness of the TMJ	N/A	N/A	N/A	N/A	N/A	N/A	7.69 (a) 3.85 (c)	0.00 (a) 3.85 (c)	N/A	N/A	N/A	N/A	N/A	N/A	N/A	N/A	6.7
Frequent jaw dislocations	N/A	N/A	N/A	N/A	N/A	N/A	3.85	0.00	N/A	N/A	N/A	N/A	N/A	N/A	N/A	N/A	N/A
Difficulties opening the mouth	N/A	N/A	N/A	N/A	N/A	N/A	3.85	0.00	N/A	N/A	N/A	N/A	N/A	N/A	N/A	N/A	N/A
a: symptoms recorded anamnestically; c: symptoms recorded clinically

**Table 4.** Frequency of symptoms among subjects suffering from TMD

	**First author [reference]**																
	Barros [7]		Weiler [8]		Weiler [9]		Persson [10]		Mendoza-Puente [11]		Bonotto [12]				Zamora- Olave [13]	Zamora- Olave [14]	Gay-Escoda [15]
	Anabolic steroids	No anabolic steroids	Basketball players	Non-athlete	Basketball/ Handball players	Non-athletes	Wrestlers	Nonwrestlers	Boxers	Handball players	Competitive karatekas	Amateur karatekas	Competitive MMA athletes	Non-athletes	Field hockey players	Water polo players	Pro soccer players
**TMD-symptoms (prevalence among all subjects in %):**																	
Tenderness on palpation of the masticatory muscles	N/A	N/A	66.8	60.0	33.33	50.00	N/A	N/A	N/A	N/A	23.1	33.3	50.0	50.0	N/A	N/A	N/A
Bruxism	N/A	N/A	50.0	0.0	26.67	12.50	N/A	N/A	N/A	N/A	N/A	N/A	N/A	N/A	N/A	N/A	N/A
Otalgia	N/A	N/A	25.0	20.0	20.00	12.50	N/A	N/A	N/A	N/A	N/A	N/A	N/A	N/A	N/A	N/A	N/A
Ear noise	N/A	N/A	25.0	20.0	13.33	12.50	N/A	N/A	N/A	N/A	N/A	N/A	N/A	N/A	N/A	N/A	N/A
Unilateral chewing	N/A	N/A	8.3	40.0	14.29	12.50	N/A	N/A	N/A	N/A	N/A	N/A	N/A	N/A	N/A	N/A	N/A
Mandibular deviation	N/A	N/A	25.0	20.0	6,67	25.00	N/A	N/A	N/A	N/A	N/A	N/A	N/A	N/A	N/A	N/A	N/A
headache	N/A	N/A	25.9	20.0	20.00	12.50	N/A	N/A	N/A	N/A	N/A	N/A	N/A	N/A	N/A	N/A	N/A
Crepitations of the TMJ	N/A	N/A	16.6	20.0	26.67	25.00	N/A	N/A	N/A	N/A	N/A	N/A	N/A	N/A	N/A	N/A	N/A
Disc displacement	N/A	N/A	N/A	N/A	N/A	N/A	N/A	N/A	N/A	N/A	84.6	66.7	62.5	50.0	N/A	N/A	N/A
Tenderness of the TMJ	N/A	N/A	N/A	N/A	N/A	N/A	N/A	N/A	N/A	N/A	N/A	N/A	N/A	N/A	N/A	N/A	N/A
Frequent jaw dislocations	N/A	N/A	N/A	N/A	N/A	N/A	N/A	N/A	N/A	N/A	N/A	N/A	N/A	N/A	N/A	N/A	N/A
Difficulties opening the mouth	N/A	N/A	N/A	N/A	N/A	N/A	N/A	N/A	N/A	N/A	N/A	N/A	N/A	N/A	N/A	N/A	N/A

## Discussion

### Inconsistencies of individual studies

The study by Weiler et al. [8] shows discrepancies between the percentage values for crepitations listed in the “Results” section and those found in the “Discussion” section. The present review adopted the value from the “Results” section for the group of non-athletes, as this was the only way to convert the percentage figure into an even number of persons [8]. The percentage value for the athlete group had to be taken from the “Discussion” section, as this information was not provided in the “Results” section [8].

In the other publication by Weiler et al. [9], the values for deviation and bruxism in the written results text do not match those in the table. This literature review refers to the data shown in the table [9]. In the athlete group, the prevalence value for headache as a symptom differs between the results text and the discussion: while the results part indicates that 20% (= three subjects) of all athletes are diagnosed with TMD report headache as a symptom, the discussion text states that it is only 13.33% (= two subjects) [9]. In relation to the entire group of athletes, this is a difference of 3.37% (3/89) to 2.25% (2/89) [9]. With regard to the study by Persson et al. [10], which reports headache in 3.85% of the cases, this literature review adopted the value presented in the “Results” section, stating a headache prevalence of 3.37% [9].

In the frequency data taken from the study by Mendoza-Puente et al. [11], the addition of “at least moderate TMD” (see Table 2) was necessary as this study does not clearly specify the difference between a healthy subject and one with a mild form of temporomandibular dysfunction. While the “Methods” section indicates that values smaller than 5 were considered healthy, the table in the “Results” section suggests that values smaller than 5 were interpreted as a mild dysfunction according to the clinical Helkimo index [11]. This would correspond to a TMD prevalence of 100%, so only moderate and severe dysfunctions according to the clinical Helkimo index were taken into account for the present review [11].

Bonotto et al. [12] examined the subjects using the RDC/TMD. In the present literature review, only Axis I was considered, since no comparative values for Axis II were available from other sources than the study by Bonotto et al. [12]. One of the charts in the study (page 283, Fig. 1) “Prevalence of temporomandibular disorders (TMD) in the different groups according to Axis I of the RDC/TMD” [12]) reports a 65.1% prevalence of TMD among competitive mixed martial arts athletes [12]. Since the relative number of healthy persons was 38.5%, the adopted prevalence of sick subjects for this review was 61.5% [12], since this was the only way to calculate an even number of individuals.

### Prevalence of temporomandibular dysfunctions in athletes

Due to different methodological approaches regarding various study parameters (e.g., collection of clinical or anamnestic data [11, 13], evaluation of different symptoms [12, 15]) and partly missing data on the performance level of the investigated groups [7, 10, 13, 14], the resulting values of the studies can only be compared to a limited extent. For example, Bonotto et al. [12] found TMD frequencies of 54.2% (competitive karateka) and 61.5% (competitive MMA athletes) in their athlete groups and Mendoza-Puente et al. [11] found 77.77% (boxers) and 45.00% (handball players) in similar case numbers, while Zamora-Olave [13] reported TMD in only 11.7% (field hockey players) of their athletes in significantly larger case numbers. The TMD prevalences in non-athletes on the other hand differ less drastically across the various studies: values between 11.11% [9] and 14.3% [12] were noted. The values according to Heß [4] (17.1%) and the anamnestically determined values of the third German oral health study [5] (21.3%) can also be classified in this order of magnitude, while Barbosa et al. [3] reported a significantly higher percentage (39.3%). The prevalence value of 17.6% in a group of recreational athletes (amateur karateka) was close to that of inactive subjects [8, 9, 12].

In the various studies, the diagnosis of TMD was defined based on different symptoms.

#### Crepitations in the TMJ

A total of five studies [8–10, 12, 15] clinically determined the relative frequency of crepitations in the TMJ. Again, however, the values of the athlete groups differ greatly. Bonotto et al. [12] identified the highest prevalences by far, stating 45.8% for competitive sports karateka and 38.5% for competitive MMA athletes. Gay-Escoda et al. [15] (16.7%, professional soccer players) and Persson et al. [10] (15.38%, wrestlers) determined comparable values in approximately equivalent group sizes (30 soccer players, 26 wrestlers). However, Gay-Escoda et al. [15] relied on clinical data, while Persson et al. [10] determined these values anamnestically. Weiler et al. [8] and Weiler et al. [9] presented considerably lower values in the studies. Both the male and female groups of athletes displayed values around 4% (4.3%, male basketball players; 4.49%, female basketball and handball players) [8, 9], which can possibly be attributed to the identical approach in both studies [8, 9]. The clinically determined value of 3.85% (wrestlers) by Persson et al. [10] also fits into this range. The corresponding values of the non-athletes are considerably lower than the frequencies of the respective athlete groups (Weiler et al. [8]: 2.4%, Weiler et al. [9]: 2.78%, Bonotto et al. [12]: 7.1%). In the third German oral health study [5], the determined values were noticeably higher: crepitations in the TMJ were clinically evident in 33.0% of adults. If one compares only the prevalence of symptoms in the group of patients, the values of non-athletes (50.0%) presented by Bonotto et al. [12] are roughly equivalent to those of Osiewicz et al. [2] (48.9%) and Manfredini et al. [16] (54%) for a group of TMD patients. However, both of the latter examined significantly larger case numbers.

#### Masticatory muscles

Three studies provide information on the prevalence of pain in the masticatory muscles [8, 9, 12]. The highest rate, 30.8% (competitive MMA athletes), is found in the study by Bonotto et al. [12]. The values determined for competitive karateka and basketball players are roughly equivalent at 12.5% [12] and 17.4% [8]. Pain in the masticatory muscles was less frequent in female basketball and handball players (5.62%) [9]. Non-athletes displayed comparably low values in all three studies (7.3% [8], 5.56% [9], and 7.1% [12]). These values, however, are still higher than the clinically and anamnestically determined prevalence of TMD in the third German oral health study (1.9% and 1.8%, respectively) [5]. Soares et al. [17] detected muscle afflictions in young adults with a prevalence of 25.7%. This percentage is in the same range as those of the athlete groups included in this review. Regarding the frequency distribution among non-athletes suffering from TMD, one can find similarly high results in the data of Osiewicz et al. [2]: 60.0% [8], 50.0% [9, 12], and 56.9% [2]. It needs to be stressed, however, that the extreme difference in case numbers limits comparability.

#### Overall

Generally, regarding both the occurrence of individual symptoms as well as the prevalence of a manifest temporomandibular dysfunction, the same pattern can be observed: athletes obviously have a greater tendency to develop TMD and tend to show aggravated symptoms. This permits the conclusion that competitive athletes are exposed to greater stress, whether due to the increased risk of injury [9, 10, 12, 14], the high training intensity [12], or the psychological pressure caused by the increased training effort and competitions [8, 9, 18], which in turn contributes to the development of temporomandibular dysfunctions. The study by Bonotto et al. [12] supports the conclusion that the aspect of competition in particular plays a decisive role in the development of symptoms in athletes. In comparison, recreational athletes showed significantly lower values than competitive athletes [12]. The fact that there is a correlation between psychoemotional stress and the development of temporomandibular dysfunctions has also been proven in studies by Kanehira et al. [19] and Wieckiewicz et al. [20].

### Evaluation of the studies

The evaluated studies refer to different sports, all of which are contact sports. These frequently lead to injuries, also in the orofacial area [21, 22], which can promote the development of temporomandibular dysfunctions [23]. This can explain, for example, the high values of boxers (77.77% [11]) or competitive MMA athletes (61.5% [12]). It would be interesting in this context to draw a comparison to non-contact-sports, such as swimming, track and field athletics, or triathlon. Future studies should take this into account.

The training intensity and athletic performance level are not always clearly specified in the studies (Table 1). Barros et al. [7], Persson et al. [10], and Zamora-Olave et al. [13, 14] did not define the athletic performance level or the training workload of the participating subjects. This missing information should be considered when assessing the studies, as it might lead to an information bias. Further studies should define a clear distinction between non-athletes and competitive athletes, similar to that made by Bonotto et al. [12]. The percentage of recreational athletes in the general population is high [24]. Therefore, the performance level of an athlete should be evaluated based on, for example, recent competitive behavior and should serve as a benchmark where appropriate. This provides the opportunity to filter out if and to what extent athletic activity should be recommended as a means of preventing temporomandibular dysfunctions.

The methods of examination differ greatly from one study to the next. In this context, it would be appropriate to adopt established, standardized methods such as the RDC or DC/TMD, which is approved for research purposes [25–27].

The case numbers are very small in some of the trials [7, 8, 10–12, 15]. The results of these studies are therefore less reliable and should rather serve as a first orientation for further research. Especially the study of Barros et al. [7] holds a high risk of bias due to the extremely small case number of four participants per group.

As has been shown in a number of studies [3, 5, 28], age is a key factor in the prevalence of temporomandibular dysfunction. There is a peak in young adults up to mid-age, while younger and older persons are less frequently affected [3, 5, 28]. In four of the studies included in this review [8, 9, 13, 14], the average age was below 18 years, so that the manifestation of symptoms is probably less severe than in athletes of older age groups.

A variety of studies have documented that TMD is more common in women [2–4, 28–30]. The included studies, however, examined predominantly male subjects [7, 8, 10, 11, 15]. Only one study examined TMD prevalence exclusively in women [9], while in three studies [12–14] no clear separation by gender was made. In addition to differing group sizes, the study by Bonotto et al. [12] lacks consistent gender distribution. The ratio of female subjects varies between 23.08% (competitive mixed martial arts athletes) and 29.17% (competitive karatekas) [12]. With regard to a possible selection bias, the reliability of this study is debatable. Because of the inconsistencies in the ratio of female to male subjects, the same pertains to the studies by Zamora-Olave et al. [13, 14].

Due to the mentioned inconsistencies in individual studies and the possibly increased risk of bias in some articles, the results of this review provide a first orientation in still poorly investigated field of dental medicine.

## Conclusions

To conclude, it can be stated that there are only few studies on the aspect of temporomandibular dysfunctions in competitive athletes to date. To make more detailed and significant statements, further studies are necessary. In order to ensure the highest possible quality for the results, the following should be taken into account:

- Large case numbers
- Inclusion of non-contact sports
- Classification according to strength and endurance sports
- Clear definition of a line between competitive and recreational sports
- Standardized examination methods

## Clinical relevance

Based on the results of studies on this topic that were published to date, there seems to be a trend that competitive athletes suffer from TMD more frequently. As the techniques applied in competitive sports are constantly refined to further improve performance, the effects on the masticatory system should be increasingly considered as well. This enables athletes to take preventive measures at an early stage to protect themselves from possible negative effects on the orofacial system and the resulting reduction in athletic performance.

## Data Availability

All data generated or analyzed during this study is included in this published article and its supplementary information files.

## Abbreviations

a: symptoms recorded anamnestically
A_i_O, A_i_I, A_i_II: anamnestic Helkimo index
c: symptoms recorded clinically
d: days
D_i_O, D_i_I, D_i_II: clinical Helkimo index
DC/TMD: diagnostic criteria for temporomandibular disorders
et al.: and others
f: female
fig.: figure
h: hours
incl.: inclusive
m: male
MMA: mixed martial arts
n: case number
N/A: not available
RDC/TMD: research diagnostic criteria for temporomandibular disorders
TMD: temporomandibular dysfunctions
TMJ: temporomandibular joint
